# Impact of contralateral pelvic drop and femoral adduction on the femoral head acetabular coverage: A study on the reproducibility of a new radiographic measurement method

**DOI:** 10.1002/jeo2.70215

**Published:** 2025-04-01

**Authors:** Renato Locks, Eliane C. Guadagnin, Guilherme Pradi Adam, Felipe F. Gonzalez, Jorge Chahla, Liszt Palmeira de Oliveira, Leonardo Metsavaht, Gustavo Leporace

**Affiliations:** ^1^ Instituto Brasil de Tecnologias da Saúde Rio de Janeiro Brazil; ^2^ Escola Paulista de Medicina Universidade Federal de São Paulo Brazil; ^3^ Clínica Imagem, Florianópolis Brazil; ^4^ RUSH University Medical Center USA; ^5^ Programa de Pós‐Graduação em Ciências Médicas Universidade do Estado do Rio de Janeiro Brazil

**Keywords:** biomechanics, borderline dysplasia, gait, femoroacetabular impingement, reliability

## Abstract

**Purpose:**

Traditional radiographic measurements for acetabular dysplasia and femoroacetabular impingement syndrome (FAIS) are typically done in static positions, overlooking dynamic behaviours. This study investigated the reproducibility of a new radiographic method that incorporates pelvic and femoral motion during running.

**Methods:**

This cross‐sectional retrospective study included 10 patients (5 males/5 females; Mean 42.4, SD: 3.0 years) with symptomatic unilateral FAIS. Participants underwent three‐dimensional running analysis and standard supine anteroposterior (AP) pelvis radiographs. Using specialised software, the femur and pelvis were rotated in the coronal plane according to peak angles of contralateral pelvic drop and femoral adduction from the running analysis, preserving the original hip joint centre. Two experienced physicians measured the lateral centre edge angle (LCEA), acetabular index (AI), sharp angle (SA), extrusion index (EI), and femoro‐epiphyseal acetabular roof index (FEAR INDEX) on both standard and manipulated (M) radiographs in two rounds, with a 15‐day interval. Differences between the original and manipulated measurements (VAR) were also calculated. Intra‐ and inter‐rater reliability were assessed using the intraclass correlation coefficient (ICC) and Bland‐Altman method, with a significance level of 5%.

**Results:**

The average contralateral pelvic drop and femoral adduction used for image manipulation were 4.7° (SD: 2.7) and 6.2° (SD: 2.4), respectively. Of the 15 radiographic measurements, 14 showed good to excellent inter‐rater reliability in the first assessment (range: 0.76‐0.98), which decreased to 11 in the second assessment (range: 0.80–0.96). Intra‐rater reliability showed 13 and 12 measurements with good or excellent reliability for raters 1 (range: 0.75–0.97) and 2 (range: 0.79–0.97), respectively.

**Conclusion:**

This study demonstrates that incorporating dynamic motion into femoral head acetabular coverage radiographic measurements provides potential reliable assessments for most parameters. Integrating motion analysis with radiography could improve understanding of acetabular coverage in active individuals and support surgical decision‐making.

**Level of evidence:**

Diagnostic Study, Level III.

**Abbreviations**
AI: acetabular index
AP: anteroposterior
CAST: calibrated anatomical systems technique
DICOM: digital imaging and communications in medicine
EI: Extrusion Index
FAIS: femoroacetabular impingement syndrome
FEAR INDEX: Femoro‐Epiphyseal Acetabular Roof Index
GRASS: Guidelines for Reporting Reliability and Agreement Studies
ICC: intraclass correlation coefficient
LCEA: lateral centre‐edge angle
MAI: Manipulated Acetabular Index
MDC: minimal detectable change
MEI: Manipulated Extrusion Index
MFEAR INDEX: Manipulated Femoro‐epiphyseal Acetabular Roof index
MLCEA: manipulated lateral centre‐edge angle
MSA: manipulated sharp angle
SA: sharp angle
SD: standard deviation
SEM: standard error of measurement
VAR AI: Variation of Acetabular Index
VAR EI: Variation of Extrusion Index
VAR FEAR INDEX: Variation of Femoro‐Epiphyseal Acetabular Roof Index
VAR LCEA: variation of lateral centre‐edge angle
VAR SA: variation of sharp angle

## INTRODUCTION

The hip joint stability relies on static and dynamic stabilisers, the bony covering of the acetabulum, labrum and capsule, and ligaments are considered static stabilisers. The negative intra‐articular pressure and muscular forces comprise the hip's dynamic stabilisers. Femoroacetabular impingement syndrome (FAIS) is a motion‐related clinical disorder of the hip with a triad of symptoms, clinical signs and imaging findings. It represents symptomatic premature contact between the proximal femur and the acetabulum [11]. Joint‐preserving surgery to treat FAIS, dysplasia, labral tears, and soft‐tissue laxity has become increasingly popular in the last decades and the understanding of all stability factors and their interactions has become mandatory [16].

The coronal plane femoral neck orientation is known to be influenced by the knee alignment in standing position [1]. On the other side, despite being considered also a static stabiliser, the bone coverage of the femoral head may be influenced by dynamics in sports or even everyday activities. Recently, measurements such as femoral head translation and pelvic tilt have been associated with distinct phenotypes of acetabular coverage, indicating the dual static and dynamic role of bone coverage of the femoral head during weight‐bearing activities [12, 22]. Running is a common recreational and competitive activity for all ages. Unfortunately, this activity is often associated with musculoskeletal injuries [21]. Running gait analysis has been utilised to evaluate patterns that are associated with several common running injuries [2, 6]. One of the main causes of injuries in the coronal plane is the dynamic knee valgus. This motion dysfunction is characterised by the medial displacement of the knee during dynamic tasks, normally caused by combinations of excessive contralateral pelvic drop, hip adduction and internal rotation [31]. While previous studies have focused on running and its impact on knee mechanics, there is limited information regarding how running influences the in vivo mechanical loading and stability of the hip joint [14, 17, 24].

Many radiographic measurements have been described and validated in the literature to assess the femoral head acetabular coverage. In the anteroposterior (AP) view of the pelvis (coronal plane), the most used are the lateral centre‐edge angle (LCEA), Acetabular Index (AI), also called Tonnis angle, the Extrusion Index (EI), sharp angle (SA) and the Femoro‐Epiphyseal Acetabular Roof (FEAR) INDEX [26, 30]. Considering the inter and intra‐rater reliability in borderline hips, the FEAR INDEX showed excellent inter‐ and intra‐rater agreement in a previous study. The inter‐ and intra‐rater reliability was fair to good for the LCEA, whereas the AI was excellent for both yet inferior to the FEAR INDEX [30]. However, all previously validated measurements are performed on standard static radiographs.

There is a lack of data regarding the influence of motion, mainly, the contralateral pelvic drop and the compensatory femoral adduction, more popularly known as Trendelenburg sign, during gait or running on the femoral head bone coverage and acetabular angles. The first step to fill this gap is to determine the reliability of the representation of dynamics in the conventional radiographic parameters previously validated in the literature. The aim of this study was to assess the intra and inter‐rater reliability of the hip measurements performed on the conventional AP pelvis and in the manipulated radiographs reproducing the pelvic drop and femoral adduction occurring during running. The authors hypothesised that reliable measurements could be obtained for the LCEA, SA, AI, EI, and FEAR INDEX across both conventional and manipulated X‐rays, as well as for the variations observed between conventional and manipulated radiographs.

## METHODOLOGY

### Participants

A convenience sample of 20 hips (bilateral assessments of 10 patients—five males and five females) with a prior diagnosis of symptomatic unilateral FAIS, who had undergone running biokinetic analysis and had standardised supine AP pelvic radiographs on record at our institution between 2020 and 2023, were invited to provide informed consent to participate in this validation study. FAIS diagnosis was defined based on the Warwick agreement [11]. The average age was 42.4 (SD: 3.0) years and the body mass index was 24.6 (SD: 0.95) kg/m^2^. This study was approved by the Universidade Federal de São Paulo Ethics Committee under the number 6.621.087.

Patients with poor‐quality radiographs according to the European guidelines on quality criteria for diagnostic radiographic images [9], previous hip or spine surgeries, signs of hip osteoarthritis and lower limb length discrepancies were excluded to avoid any factor that could influence the pelvic and lower limb biomechanics.

### Running biokinetic analysis

The running biokinetic analysis were performed in the biomotion lab utilising four high‐frequency Qualysis cameras (Göteborg, Sweden), collecting with an acquisition frequency of 250 Hz. Reflective markers were positioned on the patient body, as described in a previous study [20]. After marker placement, a static trial was performed, utilising a pointer equipped with two markers to digitise the landmarks according to the Calibrated Anatomical Systems Technique (CAST) [4], establishing the orthostatic posture of each individual for calibration and determining anthropometric and inertial parameters. After this, a functional calibration was performed to calculate hip and knee joint centres [3, 8].

The participants run on a treadmill (ProForm Pro 2000, ProForm, USA) with their regular shoes and at a standardised speed of their choice. Only whole number speeds were used, ranging from 8 km/h to 12 km/h, according to subjects' preference. After a 3‐min warmup, two trials of 1 min were collected, totalising 5 min of testing.

**Figure 1:**
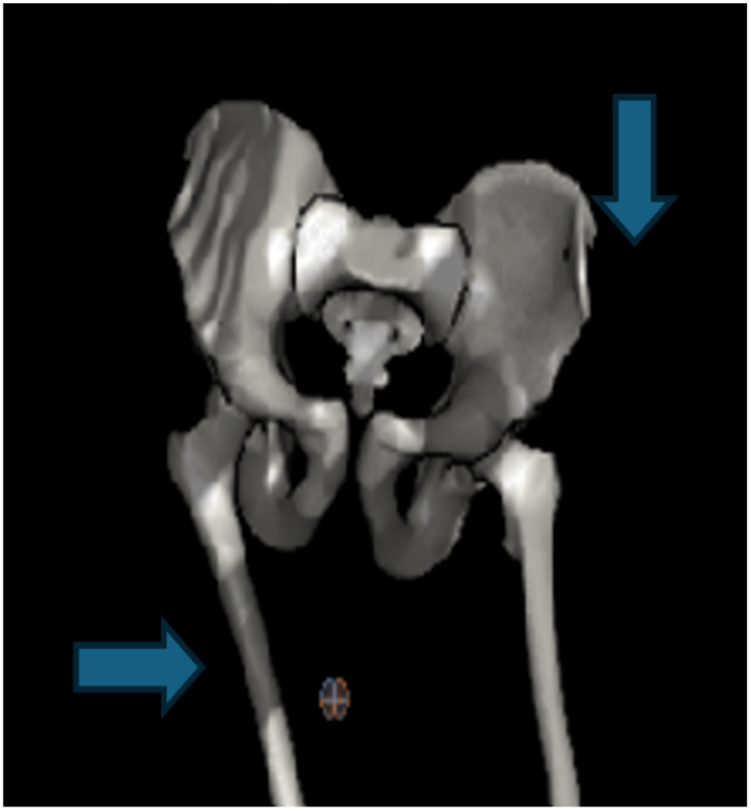
Three‐dimensional reconstruction generated from running motion analysis depicting right‐side femoral adduction and contralateral pelvic drop.

After data processing, the peak of contralateral pelvic drop for each side during the stance phase of running was determined and extracted, along with the femoral adduction at the same running stance cycle moment Figure 1. The kinematic parameters were determined bilaterally.

### Radiographic evaluation

The radiographic view was acquired with a standardised technique that was used for evaluation. The film‐focus distance was 120 cm for both views. The centre of the X‐ray beam was directed to the midpoint of the symphysis and a line connecting the anterosuperior iliac spines [9, 28]. The AP radiograph was used to calculate the radiographic parameters. The X‐ray images were numbered from 1 to 10 and shared with the investigators without any patient identification in DICOM format files.

### Image manipulation

The AP pelvis X‐ray images were uploaded, triplicated and two of the images were manipulated in the DICOM format using the Adobe Photoshop Software (Adobe Photoshop. Version 22.0.1, Adobe Systems Incorporated, 2021, USA) by an Adobe Photoshop expert, under supervision of a senior orthopaedic surgeon.

**Figure 2:**
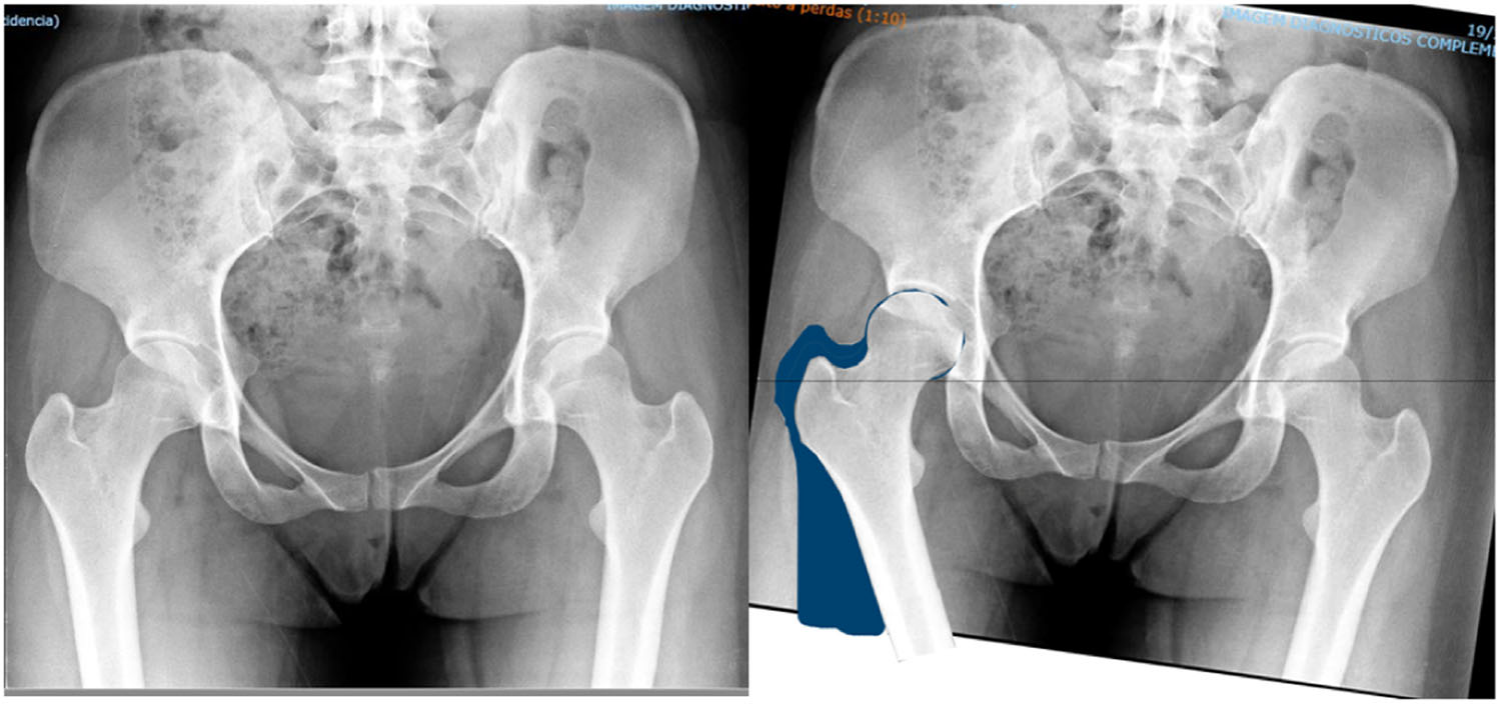
Left: Anteroposterior (AP) pelvis standard X‐ray. Right: Pelvis X‐ray depicting the image manipulation. Femur and the pelvis were rotated following the peak angles of pelvic drop and femoral adduction extracted from the coronal plane running motion analysis (right hip).

In Adobe Photoshop, a line connecting the bottom of both tears drop lines was drawn, in the sequence, the affected‐side femur was segmented from the native image and keeping the initial rotational centre of the hip joint, the femur and the pelvis were rotated following the peak angles of pelvic drop and femoral adduction extracted from the running biomotion analysis of the affected side (Figure 2). The initial teardrop line was kept and a new line was drawn at the same position of the native teardrop line. This horizontal line represents the ground surface and the respectively, body gravity centre, which is stable despite the motion between body segments. The same procedures were repeated for the non‐affected side in the remaining duplicated image. Both images were identified with patient number and side and saved in the respective patient file.

### Radiographic measurements

Using the Carestream software (Carestream Software. Version 12.0, Carestream Health, 2023, USA), the measurements described below were obtained from the supine AP pelvis X‐rays for 20 hips by a senior hip surgeon (more than 12 years of experience) and a senior musculoskeletal radiologist (more than 15 years of experience), they were identified as Rater 1 and Rater 2, respectively: All measurements adhered strictly to the guidelines provided in the original publications describing each method.

- Lateral centre edge angle (LCEA): Angle formed by a line parallel to the longitudinal pelvic axis and a line connecting the centre of the femoral head with the lateral edge of the acetabular sourcil [26].
- Acetabular index (AI): Angle formed by 1. a horizontal line (inferior margin of the pelvic teardrop) and 2. a line through the most medial point of the sclerotic zone of the acetabular roof and the lateral edge of the acetabulum (sourcil) [26].
- Sharp angle (SA): Angle formed by a horizontal line (inferior margin of the pelvic teardrop) and a line connecting the teardrop to the lateral edge of the acetabulum [26].
- Extrusion index (EI): Percentage of uncovered femoral head in comparison to the total horizontal head diameter [26].
- FEAR INDEX: the angle measured between the central part of the physeal scar of the femoral head growth plate and a line through the most medial point of the sclerotic zone of the acetabular roof and the lateral edge of the acetabulum [30].

### Measurements timeframe

A pilot round of measurements was conducted before the first official round of measurements. After non‐satisfactory interrater ICC in the pilot round—mainly for the Sharp angle, extrusion and FEAR INDEX—a refinement of the measurement process was conducted. A detailed spreadsheet outlining the measurement sequence and descriptions was created and agreed upon by both examiners. Additionally, a training session was conducted to ensure both examiners were aligned on the measurement procedures.

**Figure 3:**
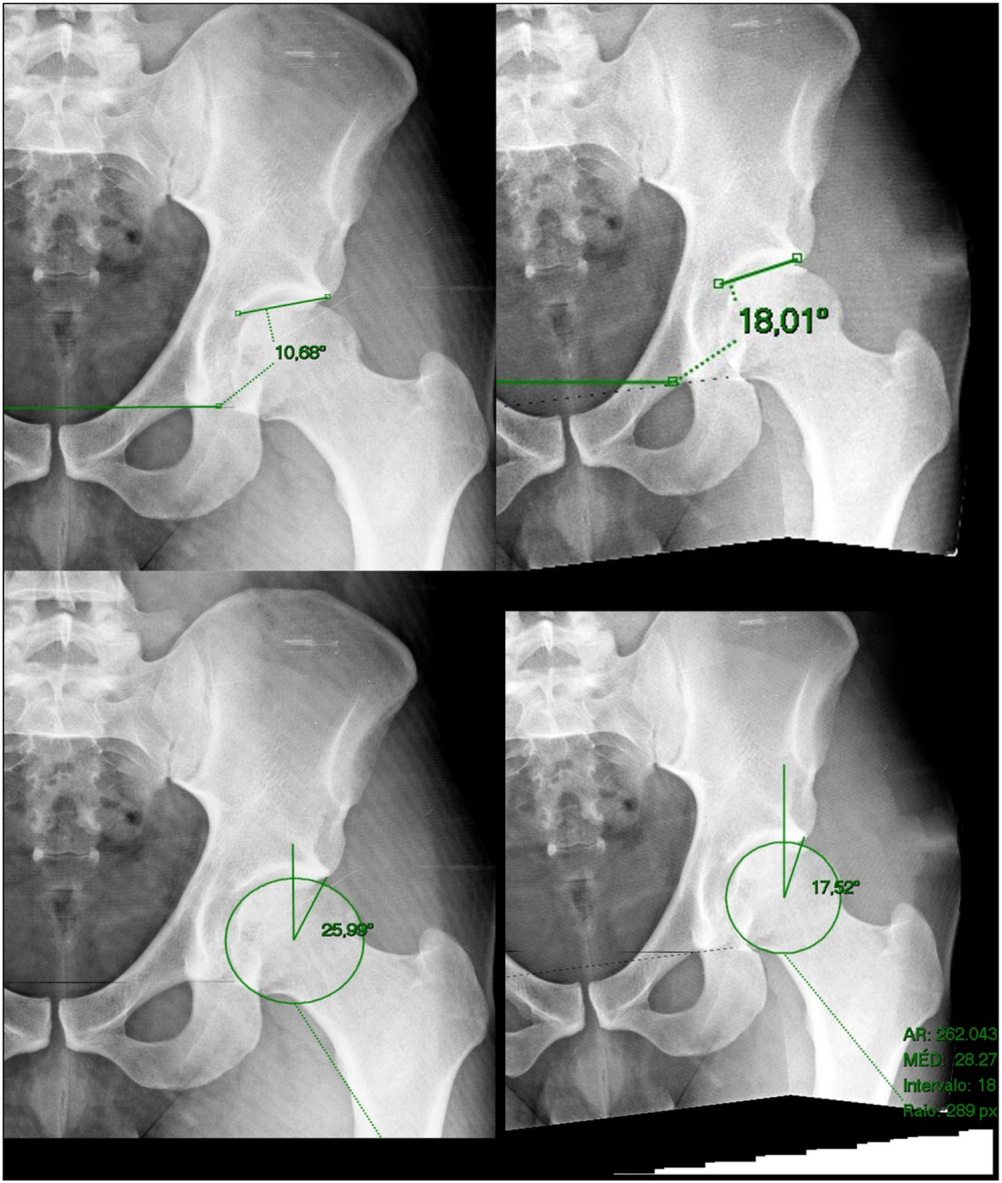
Superior images depicting the acetabular index and inferior images the lateral centre edge angle (LCEA). Left images are the original X‐rays and right images are the manipulated X‐rays.

**Figure 4:**
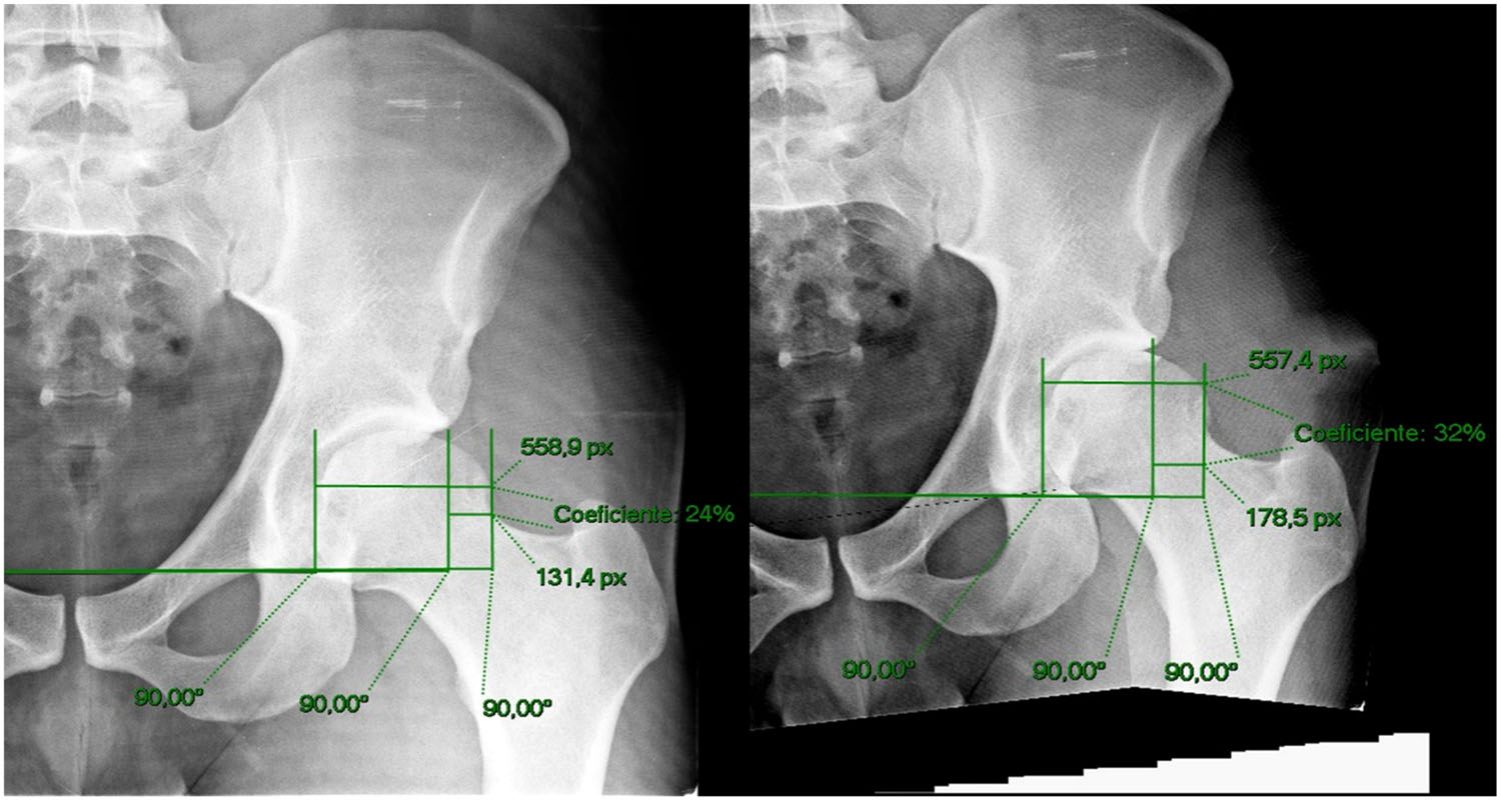
Extrusion index. Left image is the original X‐rays depicting the extrusion index (EI) and right image is the manipulated X‐ray.

**Figure 5:**
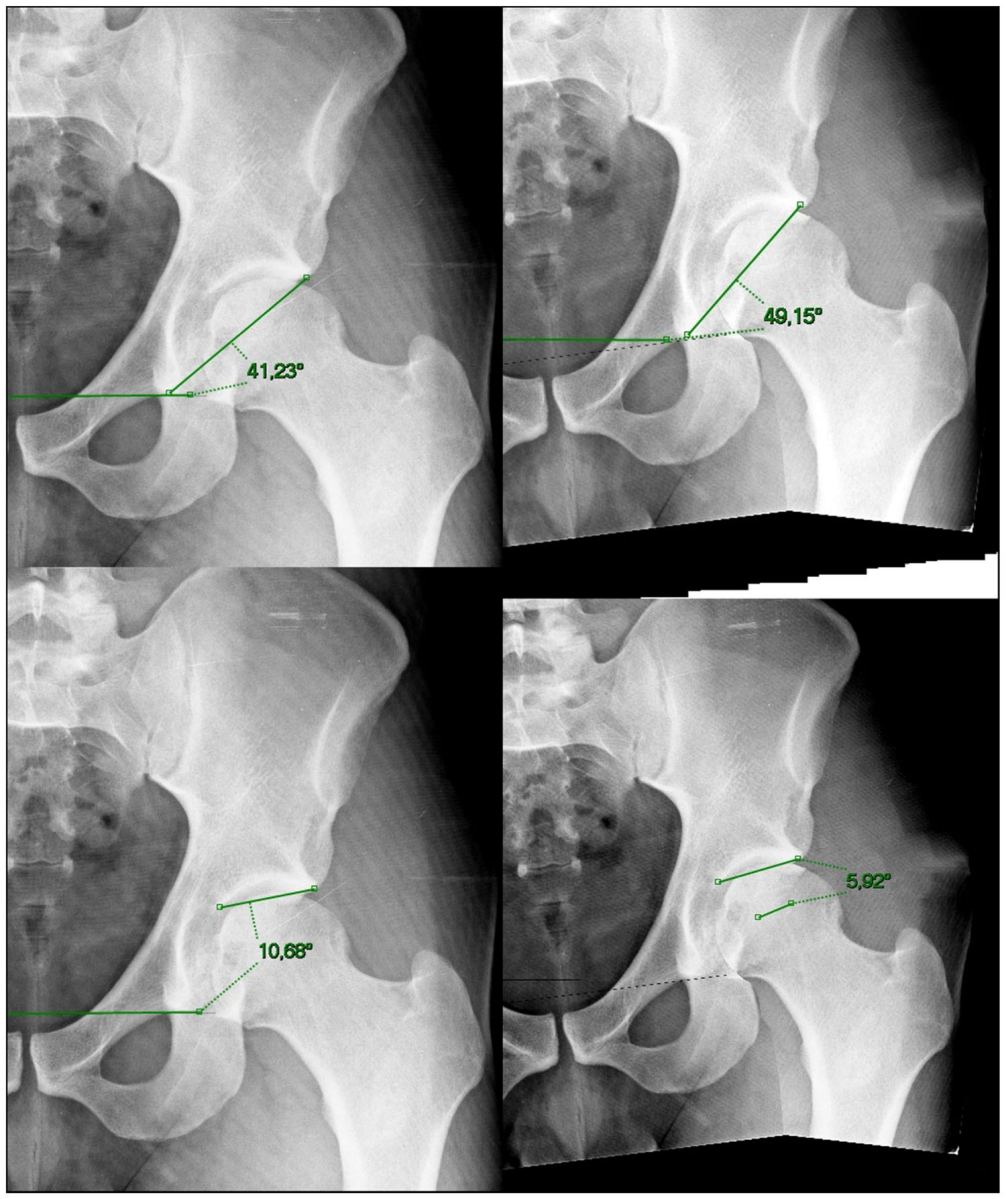
Superior images depicting the Sharp angle and inferior images the femoro‐epiphyseal acetabular roof index (FEAR Index). Left images are the original X‐rays and right images are the manipulated X‐rays.

The measurements were done independently by both examiners. First, all angles and measurements were done on the native supine AP pelvis for all 20 hips and recorded in an excel file called “native X‐ray.” Second, without looking at previous values, the same angles and measurements were performed on the right and left manipulated X‐rays, following, it was recorded in a different file called “manipulated X‐rays” (Figures 3, 4, 5). The variation index for each of the measurements was obtained by diminishing the manipulated and native X‐rays.

The first and second measurement rounds were performed 15 days apart. A third isolated round of FEAR INDEX measurements was done after a minimum of 15 days of the second round. For the intra‐rater reliability study, a second round of all measurements was performed after 15 days of the isolated FEAR INDEX round and the intraclass correlation coefficients (ICCs) calculated.

### Statistical analyses

To determine the intra and inter‐rater reliability, the ICC was determined, using a two‐way mixed effects model, based on the average of multiple measurements, with absolute agreement, within a 95% confidence interval. ICC was classified as poor for values less than 0.50, moderate for values between 0.50 and 0.75, good for values between 0.75 and 0.90, and excellent for values above 0.90 [15].

The Bland–Altman method was applied to assess agreement between measurements, with a one‐sample *t*‐test determining if differences between the raters for each assessment (inter‐rater) and between the two assessments for each rater (intra‐rater) deviated from zero. Differences between the two measurements versus their mean and limits of agreement were calculated and graphically presented. Systematic and proportional biases were examined through linear regression of measurement errors to ascertain consistency across the range of measurement errors. Differences between raters and assessments were determined using independent *t*‐tests.

The standard error of measurement (SEM) was calculated as:

$$\mathrm{SEM}={\mathrm{SD}}_{\mathrm{diff}}\times \sqrt{1-(\mathrm{ICC})}.$$

The SD_diff_ is the standard deviation of differences between measurements, defined as:

$${\mathrm{SD}}_{\mathrm{diff}}=\sqrt{\frac{\left(n_{1}-1\right)\;{\mathrm{SD}}_{1}^{2}+\left(n_{2}-1\right)\;{\mathrm{SD}}_{2}^{2}}{\left(n_{1}+n_{2}-2\right)}}.$$

The minimal detectable change (MDC), which indicates the smallest change considered to be important, was determined as:

$$\mathrm{MDC}=1.96\times \mathrm{SEM}\times \sqrt{2}.$$

For all statistical tests, a significance level of 5% was considered. The statistical software SPSS (Version 17.0, IBM Corporation, Armonk, NY, USA) was utilised for statistical analysis. All steps followed in this study were performed according to Guidelines for Reporting Reliability and Agreement Studies (GRASS) [15].

## RESULTS

Considering all 20 hips included in the study, the average pelvic drop and femoral adduction used for image manipulation were 4.7° (SD: 2.7) and 6.2° (SD: 2.4), respectively.

The inter‐rater measurements from the first round were considered satisfactory, with the exception of the FEAR INDEX. The high variability observed in this index was attributed to challenges in delineating the physis scar on the femoral head in two stable hips. A third isolated round of FEAR INDEX measurements was conducted at least 15 days after the second round, and the resulting inter‐rater ICC values were regarded as acceptable and subsequently included in the analysis.

Table 1 shows the inter‐rater analyses. For the first inter‐rater assessment, almost all measurements had an excellent or good ICC (range: 0.76–0.98), the exception was the variation of the FEAR INDEX (VAR FEAR INDEX), considered with moderate agreement (ICC: 0.62). After the second assessment, most measurements were considered excellent or good (range: 0.80–0.96), except for EI, VAR AI and VAR EI, which achieved moderate agreement (ICC: 0.74, 0.69 and 0.69, respectively), and VAR FEAR INDEX which had poor (ICC: 0.39) agreement inter‐raters.

**Table 1:** Inter‐rater analyses (first and second assessment).

Parameter	Rater 1 (mean ± SD)	Rater 2 (mean ± SD)	ICC	95% CI	SEM	MDC	*p*‐value	Interpretation
First assessment								
LCEA (°)	29.3 ± 7.0	29.5 ± 6.8	0.94	0.845–0.976	1.69	4.70	0.912	Excellent
AI (°)	3.9 ± 4.2	3.5 ± 6.0	0.94	0.857–0.977	1.24	3.43	0.799	Excellent
SA (°)	42.6 ± 3.5	41.3 ± 3.5	0.91	0.600–0.972	1.04	2.87	0.246	Excellent
EI (A.U.)	0.2 ± 0.1	0.2 ± 0.1	0.89	0.678–0.957	0.02	0.06	0.328	Good
FEAR INDEX (°)	−16.6 ± 8.0	−13.0 ± 7.1	0.89	0.364–0.967	2.51	6.95	0.148	Good
MLCEA (°)	25.1 ± 6.8	25.2 ± 7.1	0.98	0.946–0.992	1.01	2.79	0.969	Excellent
MAI (°)	9.0 ± 6.0	9.2 ± 6.5	0.98	0.937–0.990	0.98	2.73	0.906	Excellent
MSA (°)	47.4 ± 4.2	46.5 ± 4.0	0.96	0.852–0.984	0.87	2.40	0.493	Excellent
MEI (A.U.)	0.2 ± 0.1	0.2 ± 0.1	0.91	0.760–0.965	0.02	0.06	0.446	Excellent
MFEAR INDEX (°)	−7.2 ± 8.1	−3.6 ± 7.4	0.88	0.427–0.961	2.71	7.52	0.153	Good
VAR LCEA (°)	−4.2 ± 3.7	−4.4 ± 3.4	0.88	0.695–0.953	1.22	3.39	0.888	Good
VAR AI (°)	5.0 ± 4.2	5.7 ± 3.4	0.87	0.682–0.949	1.36	3.78	0.330	Excellent
VAR SA (°)	4.8 ± 2.7	5.2 ± 2.9	0.93	0.828–0.972	0.73	2.02	0.499	Excellent
VAR EI (A.U.)	0.1 ± 0.0	0.1 ± 0.0	0.76	0.378–0.904	0.02	0.04	0.805	Good
VAR FEAR INDEX (°)	9.4 ± 4.3	9.4 ± 3.3	0.62	0.020–0.853	2.36	6.54	0.974	Moderate
Second assessment								
LCEA (°)	29.8 ± 5.7	31.5 ± 5.7	0.94	0.744–0.978	1.44	4.00	0.356	Excellent
AI (°)	5.3 ± 3.4	3.8 ± 5.6	0.82	0.538–0.927	2.00	5.54	0.534	Good
SA (°)	42.5 ± 3.6	41.2 ± 3.2	0.89	0.562–0.964	1.11	3.07	0.214	Good
EI (A.U.)	0.2 ± 0.1	0.2 ± 0.1	0.74	0.364–0.896	0.03	0.09	0.521	Moderate
FEAR INDEX (°)	−17.4 ± 8.7	−15.1 ± 8.1	0.88	0.685–0.953	2.92	8.08	0.386	Good
MLCEA (°)	24.2 ± 5.6	26.2 ± 6.3	0.92	0.690–0.975	1.64	4.56	0.306	Excellent
MAI (°)	10.1 ± 5.4	8.8 ± 5.8	0.96	0.859–0.987	1.11	3.08	0.457	Excellent
MSA (°)	47.0 ± 4.6	46.0 ± 4.4	0.94	0.830–0.976	1.13	3.12	0.482	Excellent
MEI (A.U.)	0.2 ± 0.1	0.2 ± 0.1	0.94	0.860–0.978	0.02	0.05	0.637	Excellent
MFEAR INDEX (°)	−8.5 ± 9.1	−4.4 ± 9.8	0.80	0.450–0.925	4.20	11.64	0.178	Good
VAR LCEA (°)	−5.6 ± 3.0	−5.3 ± 3.1	0.81	0.505–0.923	1.35	3.74	0.783	Good
VAR AI (°)	4.7 ± 3.8	5.0 ± 3.7	0.69	0.198–0.878	2.08	5.77	0.828	Moderate
VAR SA (°)	4.5 ± 3.2	4.9 ± 4.0	0.81	0.518–0.924	1.58	4.38	0.729	Good
VAR EI (A.U.)	0.0 ± 0.0	0.1 ± 0.0	0.69	0.249–0.877	0.02	0.06	0.388	Moderate
VAR FEAR INDEX (°)	9.0 ± 3.5	10.8 ± 5.1	0.39	−0.446 to 0.751	3.45	9.56	0.211	Poor

Abbreviations: AI, acetabular index; CI, confidence interval; EI, extrusion index; FEAR INDEX, femoro‐epiphyseal acetabular roof index; LCEA, lateral centre‐edge angle; M, manipulated; MDC, minimal detectable change; SA, sharp angle; SEM, standard error of measurement; VAR, variation.

The Bland–Altman method for the inter‐rater analyses is shown in Table 2 and Supporting Information: File S1. The differences between the two raters differed from zero for EI, SA, FEAR INDEX, MSA, and MFEAR (*p* < 0.05) in the first assessment, and LCEA, SA, MLCEA, MAI, and MFEAR (*p* < 0.05) in the second. The SA and MFEAR were the only significant on both assessments. The single regression p‐value was significant only for AI in both assessments (*p* < 0.05), which indicates the existence of systematic errors for this measurement, as the AI mean moved towards zero and negative values (Supporting Information: Figures 1 and 4).

**Table 2:** Bland–Altman results for inter‐rater analyses (first and second assessment).

Parameter	One sample *t*‐test *p*‐value	Mean ± SD of the difference	Limits of agreement (lower–upper)	Single regression *p*‐value
First assessment				
LCEA (°)	0.754	−0.24 ± 3.37	−6.84 to 6.36	0.796
AI (°)	0.441	0.43 ± 2.43	−4.33 to 5.18	<0.001*
SA (°)	0.002*	1.32 ± 1.62	−1.86 to 4.51	0.853
EI (A.U.)	0.027*	0.02 ± 0.04	−0.06 to 0.10	0.530
FEAR INDEX (°)	<0.001*	−3.53 ± 3.62	−10.62 to 3.56	0.329
MLCEA (°)	0.862	−0.08 ± 2.07	−4.13 to 3.97	0.484
MAI (°)	0.603	−0.23 ± 1.98	−4.12 to 3.65	0.285
MSA (°)	0.013*	0.91 ± 1.49	−2.01 to 3.83	0.511
MEI (A.U.)	0.057	0.02 ± 0.04	−0.06 to 0.10	0.607
MFEAR INDEX (°)	0.001*	−3.57 ± 4.12	−11.65 to 4.51	0.482
VAR LCEA (°)	0.767	0.16 ± 2.35	−4.45 to 4.77	0.595
VAR AI (°)	0.260	−0.66 ± 2.55	−5.65 to 4.33	0.171
VAR SA (°)	0.198	−0.42 ± 1.39	−3.14 to 2.31	0.516
VAR EI (A.U.)	0.699	0.00 ± 0.03	−0.06 to 0.05	0.228
VAR FEAR INDEX (°)	0.965	−0.04 ± 4.07	−8.02 to 7.94	0.184
Second assessment				
LCEA (°)	0.005*	−1.68 ± 2.37	−6.32 to 2.96	0.888
AI (°)	0.077	1.47 ± 3.52	−5.43 to 8.38	0.002*
SA (°)	0.003*	1.34 ± 1.76	−2.11 to 4.79	0.295
EI (A.U.)	0.066	0.02 ± 0.05	−0.07 to 0.11	0.163
FEAR INDEX (°)	0.060	−2.33 ± 5.20	−12.53 to 7.87	0.621
MLCEA (°)	0.004*	−1.95 ± 2.66	−7.17 to 3.27	0.205
MAI (°)	0.009*	1.22 ± 1.87	−2.45 to 4.88	0.377
MSA (°)	0.053	0.94 ± 2.04	−3.06 to 4.94	0.657
MEI (A.U.)	0.149	0.01 ± 0.03	−0.05 to 0.07	0.397
MFEAR INDEX (°)	0.017*	−4.11 ± 7.02	−17.86 to 9.65	0.645
VAR LCEA (°)	0.637	−0.27 ± 2.50	−5.17 to 4.64	0.799
VAR AI (°)	0.757	−0.26 ± 3.68	−7.47 to 6.95	0.897
VAR SA (°)	0.549	−0.40 ± 2.93	−6.14 to 5.34	0.203
VAR EI (A.U.)	0.214	−0.01 ± 0.03	−0.08 to 0.06	0.889
VAR FEAR INDEX (°)	0.158	−1.78 ± 5.40	−12.35 to 8.80	0.112

*Note*: Graphs are in Supporting Information: File S1.

Abbreviations: AI, acetabular index; EI, extrusion index; FEAR INDEX, femoro‐epiphyseal acetabular roof index; LCEA, lateral centre‐edge angle; M, manipulated; VAR, variation; SA, sharp angle; SD, standard deviation.

The intra‐rater analyses between assessments of the Rater 1 showed most of the measurements with an excellent/good reliability (ICC range: 0.75–0.97), the exception was the VAR EI considered moderate (ICC: 0.55) and VAR FEAR INDEX, poor (ICC: 0.28). The Rater 2 measurements also demonstrated excellent/good agreement (ICC range: 0.79–0.97). The exceptions were VAR LCEA, VAR EI and VAR FEAR INDEX, considered moderate (ICC: 0.72, 0.69 and 0.63, respectively; Table 3).

**Table 3:** Intra‐rater analyses (Rater 1 and Rater 2).

Parameter	First assessment (mean ± SD)	Second assessment (mean ± SD)	ICC	95% CI	SEM	MDC	*p*‐value	Interpretation
Rater 1								
LCEA (°)	29.3 ± 7.0	29.5 ± 6.8	0.93	0.817–0.971	1.72	4.78	0.801	Excellent
AI (°)	3.9 ± 4.2	3.5 ± 6.0	0.82	0.522–0.928	1.64	4.54	0.264	Excellent
SA (°)	42.6 ± 3.5	41.3 ± 3.5	0.95	0.864–0.979	0.83	2.29	0.933	Excellent
EI (A.U.)	0.2 ± 0.1	0.2 ± 0.1	0.95	0.848–0.980	0.02	0.04	0.386	Excellent
FEAR INDEX (°)	−16.6 ± 8.0	−13.0 ± 7.1	0.93	0.828–0.973	2.18	6.06	0.737	Excellent
MLCEA (°)	25.1 ± 6.8	25.2 ± 7.1	0.96	0.899–0.984	1.24	3.43	0.667	Excellent
MAI (°)	9.0 ± 6.0	9.2 ± 6.5	0.97	0.891–0.991	0.94	2.60	0.555	Excellent
MSA (°)	47.4 ± 4.2	46.5 ± 4.0	0.94	0.843–0.975	1.11	3.08	0.829	Excellent
MEI (A.U.)	0.2 ± 0.1	0.2 ± 0.1	0.96	0.721–0.988	0.02	0.04	0.388	Excellent
MFEAR INDEX (°)	−7.2 ± 8.1	−3.6 ± 7.4	0.93	0.829–0.973	2.24	6.21	0.633	Excellent
VAR LCEA (°)	−4.2 ± 3.7	−4.4 ± 3.4	0.75	0.365–0.899	1.69	4.67	0.207	Good
VAR AI (°)	5.0 ± 4.2	5.7 ± 3.4	0.85	0.605–0.939	1.57	4.34	0.816	Good
VAR SA (°)	4.8 ± 2.7	5.2 ± 2.9	0.91	0.769–0.963	0.90	2.49	0.717	Excellent
VAR EI (A.U.)	0.1 ± 0.0	0.1 ± 0.0	0.55	‐0.132–0.823	0.02	0.07	0.536	Moderate
VAR FEAR INDEX (°)	9.4 ± 4.3	9.4 ± 3.3	0.28	‐0.914–0.720	3.37	9.34	0.740	Poor
Rater 2								
LCEA (°)	29.5 ± 6.8	31.5 ± 5.7	0.90	0.690–0.961	2.02	5.60	0.328	Excellent
AI (°)	3.5 ± 6.0	3.8 ± 5.6	0.97	0.934–0.989	0.96	2.65	0.865	Excellent
SA (°)	41.3 ± 3.5	41.2 ± 3.2	0.91	0.780–0.966	0.98	2.71	0.902	Excellent
EI (A.U.)	0.2 ± 0.1	0.2 ± 0.1	0.86	0.659–0.945	0.02	0.07	0.593	Good
FEAR INDEX (°)	−13.0 ± 7.1	−15.1 ± 8.1	0.95	0.774–0.982	1.76	4.87	0.390	Excellent
MLCEA (°)	25.2 ± 7.1	26.2 ± 6.3	0.96	0.903–0.986	1.28	3.54	0.634	Excellent
MAI (°)	9.2 ± 6.5	8.8 ± 5.8	0.97	0.927–0.988	1.05	2.90	0.829	Excellent
MSA (°)	46.5 ± 4.0	46.0 ± 4.4	0.97	0.920–0.987	0.75	2.08	0.731	Excellent
MEI (A.U.)	0.2 ± 0.1	0.2 ± 0.1	0.94	0.852–0.978	0.02	0.05	0.558	Excellent
MFEAR INDEX (°)	−3.6 ± 7.4	−4.4 ± 9.8	0.95	0.880–0.981	1.90	5.28	0.780	Excellent
VAR LCEA (°)	−4.4 ± 3.4	−5.3 ± 3.1	0.72	0.305–0.886	1.74	4.82	0.372	Moderate
VAR AI (°)	5.7 ± 3.4	5.0 ± 3.7	0.79	0.486–0.917	1.62	4.48	0.537	Good
VAR SA (°)	5.2 ± 2.9	4.9 ± 4.0	0.88	0.690–0.951	1.23	3.40	0.749	Good
VAR EI (A.U.)	0.1 ± 0.0	0.1 ± 0.0	0.69	0.185–0.877	0.02	0.05	0.962	Moderate
VAR FEAR INDEX (°)	9.4 ± 3.3	10.8 ± 5.1	0.63	0.100–0.851	2.62	7.26	0.340	Moderate

Abbreviations: AI, acetabular index; CI, confidence interval; EI, extrusion index; FEAR INDEX, femoro‐epiphyseal acetabular roof index; M, manipulated; LCEA, lateral centre‐edge angle; MDC, minimal detectable change; SA, sharp angle; SD, standard deviation; SEM, standard error of measurement; VAR, variation.

When the intra‐rater Bland–Altman was calculated, the differences between the two assessments differed from zero for AI, EI, MAI, MEI, and VAR LCEA for Rater 1, and for the LCEA and FEAR for Rater 2. Considering the systematic errors, LCEA was the only measurement significant for Rater 1 (as the MLCEA mean increased, the difference between the first and the second assessments was increased; Supporting Information: Figure 8). For Rater 2, systematic errors occurred for MFEAR, VAR SA and VAR FEAR Rater 2 (increased mean values, increased differences between the assessments; Table 4 and Supporting Information: Figures 11 and 12).

**Table 4:** Bland–Altman results for the intra‐rater analyses (Rater 1 and Rater 2).

Parameter	One sample *t*‐test *p*‐value	Mean ± SD of the difference	Limits of agreement (lower–upper)	Single regression *p*‐value
Rater 1				
LCEA (°)	0.509	−0.51 ± 3.37	−7.12 to 6.10	0.105
AI (°)	0.044*	−1.37 ± 2.84	−6.93 to 4.19	0.224
SA (°)	0.799	0.09 ± 1.64	−3.12 to 3.31	0.948
EI (A.U.)	0.035*	0.01 ± 0.03	−0.04 to 0.07	0.244
FEAR INDEX (°)	0.359	0.89 ± 4.24	−7.42 to 9.20	0.461
MLCEA (°)	0.119	0.85 ± 2.33	−3.71 to 5.41	0.018*
MAI (°)	0.006*	−1.08 ± 1.57	−4.15 to 2.00	0.138
MSA (°)	0.380	0.44 ± 2.17	−3.81 to 4.68	0.490
MEI (A.U.)	0.001*	0.02 ± 0.02	−0.03 to 0.07	0.440
MFEAR INDEX (°)	0.186	1.31 ± 4.27	−7.06 to 9.68	0.291
VAR LCEA (°)	0.047*	1.36 ± 2.87	−4.26 to 6.97	0.266
VAR AI (°)	0.661	0.30 ± 2.96	−5.51 to 6.10	0.561
VAR SA (°)	0.389	0.34 ± 1.72	−3.04 to 3.72	0.175
VAR EI (A.U.)	0.438	0.01 ± 0.04	−0.07 to 0.08	0.972
VAR FEAR INDEX (°)	0.720	0.42 ± 5.15	−9.68 to 10.52	0.388
Rater 2				
LCEA (°)	0.022*	−1.95 ± 3.50	−8.80 to 4.91	0.162
AI (°)	0.451	−0.32 ± 1.88	−4.00 to 3.36	0.376
SA (°)	0.798	0.11 ± 1.92	−3.65 to 3.87	0.455
EI (A.U.)	0.090	0.01 ± 0.03	−0.05 to 0.08	0.097
FEAR INDEX (°)	0.004*	2.09 ± 2.87	−3.54 to 7.72	0.150
MLCEA (°)	0.071	−1.02 ± 2.37	−5.67 to 3.63	0.156
MAI (°)	0.436	0.38 ± 2.11	−3.75 to 4.50	0.186
MSA (°)	0.167	0.47 ± 1.45	−2.38 to 3.31	0.282
MEI (A.U.)	0.070	0.01 ± 0.03	−0.05 to 0.07	0.387
MFEAR INDEX (°)	0.366	0.77 ± 3.74	−6.55 to 8.10	0.002*
VAR LCEA (°)	0.185	0.93 ± 3.03	−5.01 to 6.87	0.682
VAR AI (°)	0.300	0.70 ± 2.93	−5.05 to 6.44	0.648
VAR SA (°)	0.504	0.36 ± 2.34	−4.22 to 4.93	0.025*
VAR EI (A.U.)	0.946	0.00 ± 0.03	−0.06 to 0.06	0.220
VAR FEAR INDEX (°)	0.200	−1.32 ± 4.43	−10.01 to 7.38	0.033*

*Note*: Graphs are in Supporting Information: File S1.

Abbreviations: AI, acetabular index; EI, extrusion index; FEAR INDEX, femoro‐epiphyseal acetabular roof index; LCEA, lateral centre‐edge angle; M, manipulated; SA, sharp angle; SD, standard deviation; VAR, variation.

## DISCUSSION

The main finding of this study is that the most common radiographic measurements for assessing femoral head acetabular coverage demonstrate good to excellent inter and intra‐rater reliability when manipulated to reproduce the effects of pelvic drop and femoral adduction during running. Among the 15 investigated radiographic measurements, 14 demonstrated good to excellent inter‐rater reliability in the first assessment and that number was reduced to 11 measures during the second assessment. The intra‐rater evaluation showed 13 and 12 measurements with excellent or good reliability for Rater 1 and 2, respectively. This data confirms the potential reliability of most of the radiographic measurements representing the impact of the pelvic drop and femoral adduction during running on the manipulated AP pelvis X‐ray. However, it is noteworthy that some measurements exhibited poor to moderate reliability. The VAR FEAR INDEX had the lowest reliability in both assessments intra and inter‐rater evaluation.

As shown in a previous study, the following parameters can be reliably measured without necessarily compensating for pelvic tilt and rotation on an AP pelvic radiograph: LCEA angle, Sharp angle, acetabular and extrusion index [25]. Tannast et al. [27] previously validated a computer programme called “Hip2 Norm” that enabled the calculation specific radiographic parameters for FAIS on AP pelvic radiographs with respect to individual pelvic tilt and rotation. A good to very good reliability and reproducibility were found for LCEA, AI and EI. Another study evaluated the reliability and agreement of a newly developed artificial intelligence algorithm for the evaluation of pelvic radiographs [23]. The artificial intelligence software provided reliable results in 94.3%. The ICC values ranged between 0.73 for the Acetabular Index to 0.80 for the LCEA. Artificial intelligence‐powered applications were considered a reliable alternative to manual evaluation of pelvic radiographs. The current study confirmed the good to excellent intra and inter‐rater reliability obtained on the previous studies with standard AP pelvis radiographs. Additionally, to our knowledge, it was the first study to introduce and verify the reliability of the radiographic measurements on the supine AP Pelvis after manipulations reproducing the influence of the pelvic drop and femoral adduction on acetabular coverage during running, in the FAIS population. The variations of measurements were those that presented the worst reliability, probably related to their smaller values with proportionally high standard deviations. This may be associated with a sum of errors in the intra and inter‐analysis of the conventional parameters plus the manipulated parameters. Radiographic measurements can be used for diagnosis and decision‐making in these patients but must be used in adjunct with patient history, clinical findings, and the femoral morphology.

Uemura et al. [29] used dual fluoroscopy and 3D models extracted from computed tomography scans to compare acetabular bone coverage in the supine position and during gait in asymptomatic patients. The study showed little variation in total femoral head coverage during the gait cycle. While the authors found significant differences on a regional basis when comparing coverage during standing with that during gait, the magnitude of these differences was quite small, and likely not clinically relevant [29]. Differently, in the present study, patients with at least one symptomatic FAIS hip were evaluated, and the activity selected for comparison was running instead of walking. Although the difference between the original and manipulated images was, on average, small (<2°) across all parameters, like Uemura et al. [29], the upper and lower limits of the confidence interval of the present study were very high, reaching 10° in some radiographic measurements. The important variability in the difference between the dynamic and static measurements in this study may represent a clinically important difference for some patients who demonstrated more extreme values in terms of pelvic drop and pelvic adduction. Future studies with larger samples would be important to verify the clinical relevance of these differences on clinical decision‐making, particularly in subgroups that exhibit substantial variations in acetabular coverage between static and dynamic activities.

The clinical significance of biomechanical alterations, such as pelvic drop and femoral adduction, is well‐documented, as these factors are closely linked to different types and mechanisms of orthopaedic injuries. A cross‐sectional study involving 149 running‐injured adolescents showed that patients with bony injuries, when compared to soft tissue injuries, had significantly higher proportion of runners exhibiting contralateral pelvic drop at midstance (*χ* ^2^ = 5.3, *p* = 0.02). According to the literature, the maximum pelvic drop angle during walking in healthy controls is on average < 3° [7]. Therefore, patients with maximum pelvic drop angle ≤ 3° during walking do not adopt compensatory gait and have stable position of centre of gravity, thus do not affect the arm of ground reaction forces in the lower limb. In contrast, for patients with maximum pelvic drop angle > 3° degrees during walking, compensatory gait will lead to changes of hip and knee adduction moment, potentially leading to injury [19]. In this study, the average pelvic drop and femoral adduction used for image manipulation were 4.7° (SD: 2.7) and 6.2° (SD: 2.4), respectively, indicating a high prevalence of increased pelvic drop and femoral adduction in this study population. These elevated values are likely due to the higher physical demands of running compared to walking, which may also contribute to greater changes in acetabular coverage.

Some studies have been published looking specifically at the results of surgical treatment of borderline dysplastic hips, with one study showing higher rates of failure in the borderline hips than in those with adequate acetabular cover [13], and the other study showing comparable outcomes [18]. A recent systematic review presented the most common causes, diagnostic features, treatment options and outcomes of patients with hip micro‐instability. The most common features for diagnosis of micro‐instability on history were anterior pain in 146 (78%) patients and a feeling of instability with gait in 143 (81%) patients [5]. Specifically, for the FAIS associated with borderline dysplasia or potentially unstable hips, the dynamic radiographic measurements representing the impact of the pelvic drop and femoral adduction can be paramount in the decision of whether the hips have more characteristics of dysplasia or impingement.

This study has limitations that should be considered. First, studies involving kinematic measurements can be subject to alterations due to errors in marker placement on the skin. Second, only the pelvic drop and adduction peak angles on the coronal plane were used as the reference for image manipulation, no movement variation on the axial and sagittal plane was considered. However, radiographic measurements in a clinical setting are only possible when there is no deviation on the axial and sagittal planes. Third, this study methodology was unable to reproduce the hip translation on the radiographic manipulations. No reference regarding femoral translation during running was found in the literature search, however, a previous study on asymptomatic patients reported that hip translation during gait was only 0.6 mm, showing that accounting for translations possibly changes the results on the MLCEA and MEI [10]. Fourth, the study methodology focuses exclusively on utilising coronal plane data from the 3D analysis to align with preexisting radiographs and it's considered and starting of a new research field. Future studies considering changes in all three dimensions are necessary, however, the actual approach avoided additional radiation exposure. Fifth, only recreational runners with unilateral symptomatic FAIS were included, however, the pelvic drop and adduction were present in both symptomatic and asymptomatic sides. Lastly, due to the limited sample size and the specific characteristics of the sample, the results cannot be fully transferred to the general population with FAIS.

## CONCLUSION

This study demonstrates that a new method incorporating dynamic motion data into hip radiographic measurements is potentially reliable for most parameters. This method was able to show that femoral head bony cover varies during running. Integrating motion analysis with radiography could enhance understanding of acetabular coverage in active individuals and aid in surgical decision‐making. These results should be utilised with caution when considering populations with different characteristics.

## AUTHOR CONTRIBUTIONS

All authors contributed to the study's conception and design. Material preparation, data collection, and analysis were performed by Renato Locks, Eliane Celina Guadagnin, Guilherme Pradi Adam, Felipe Fernandes Gonzalez, and Gustavo Leporace. The first draft of the manuscript was written by Renato Locks, Eliane Celina Guadagnin, Felipe Gonzalez, Liszt Palmeira de Oliveira, Leonardo Metasvaht, and Jorge Chahla. All authors commented on previous versions of the manuscript. All authors read and approved the final manuscript.

## CONFLICTS OF INTEREST STATEMENT

Jorge Chahla reports a relationship with American Orthopaedic Society for Sports Medicine: Board or committee member; Arthrex, Inc: Paid consultant; Arthroscopy Association of North America: Board or committee member; CONMED Linvatec: Paid consultant; International Society of Arthroscopy, Knee Surgery, and Orthopaedic Sports Medicine: Board or committee member; Ossur: Paid consultant; Smith & Nephew: Paid consultant; Paid presenter or speaker. The other authors declare no conflicts of interest.

## ETHICS STATEMENT

This study was approved by the Universidade Federal de São Paulo Ethics Committee under the number 6.621.087. All patients signed the informed consent form.

## Supporting information

Supporting information.

## Data Availability

The data sets used and/or analysed during the current study are available from the corresponding author on reasonable request.
