# Supplementary material for: Impact of contralateral pelvic drop and femoral adduction on the femoral head acetabular coverage: A study on the reproducibility of a new radiographic measurement method
Source: J Exp Orthop. 2025 Apr 1;12(2):e70215. doi: 10.1002/jeo2.70215 (PMC11959493; doi:10.1002/jeo2.70215)
Supplement: Supplementary file 1 — Supporting information. [file JEO2-12-e70215-s001.docx]

**Supplementary Material**

**Figure 1.** Inter-Rater Analyses (First Assessment) for the Non-Manipulated Parameters


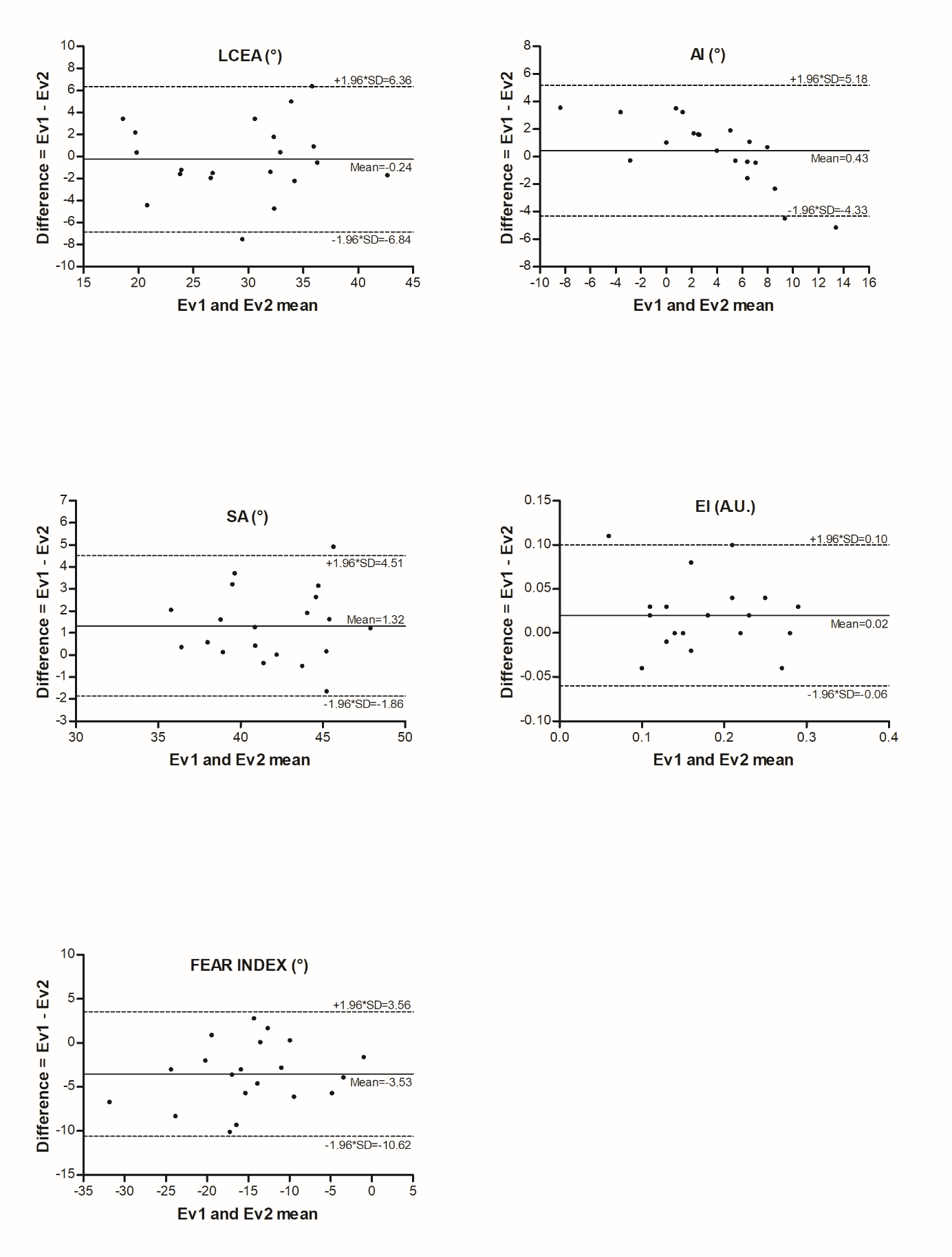


LCEA: lateral center-edge angle; AI: acetabular index; SA: sharp angle; EI: extrusion index; Ev1: Rater 1; Ev2: Rater 2.

**Figure 2.** Inter-Rater Analyses (First Assessment) for the Manipulated Parameters


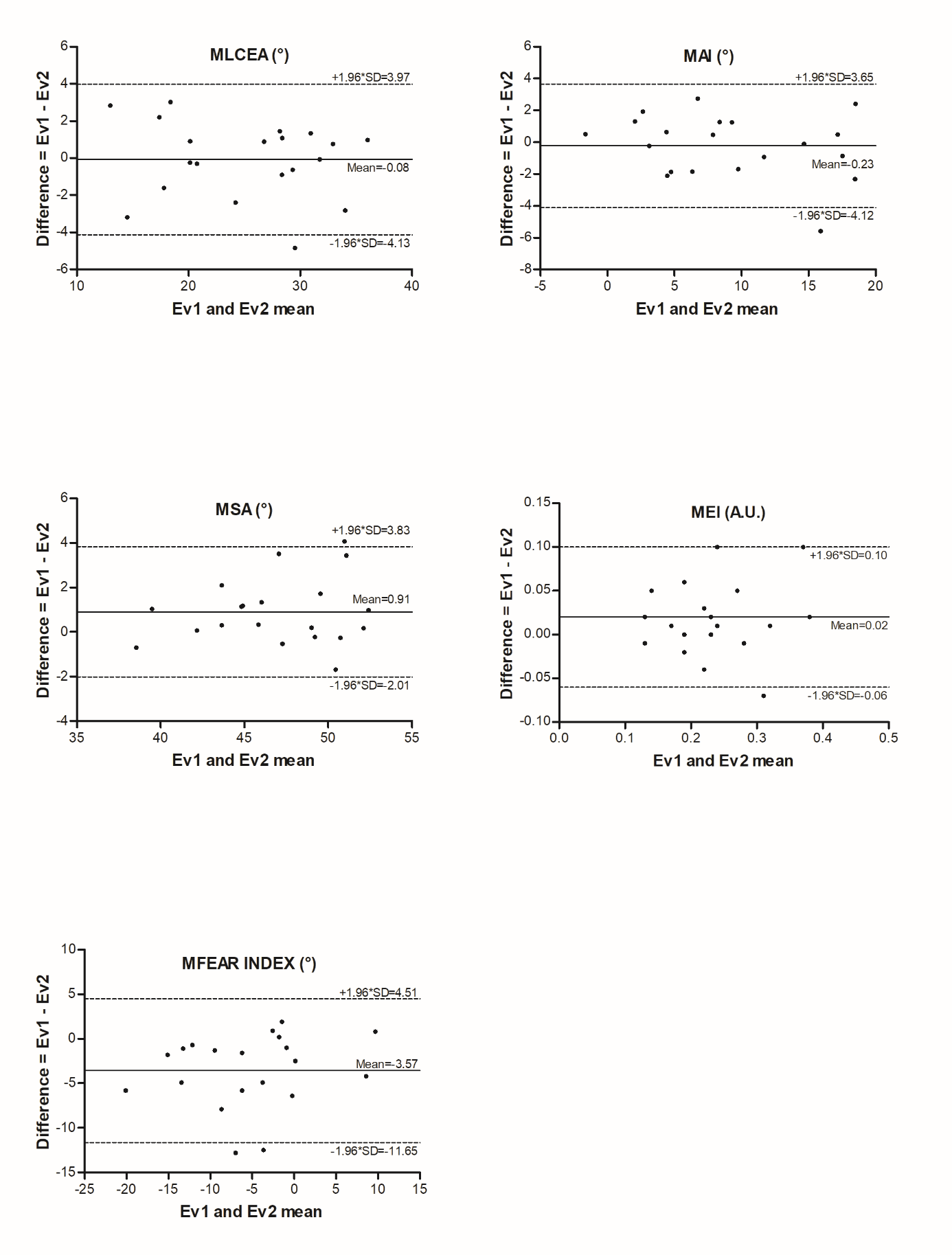


LCEA: lateral center-edge angle; AI: acetabular index; SA: sharp angle; EI: extrusion index; M: manipulated; Ev1: Rater 1; Ev2: Rater 2.

**Figure 3.** Inter-Rater Analyses (First Assessment) for the Variation


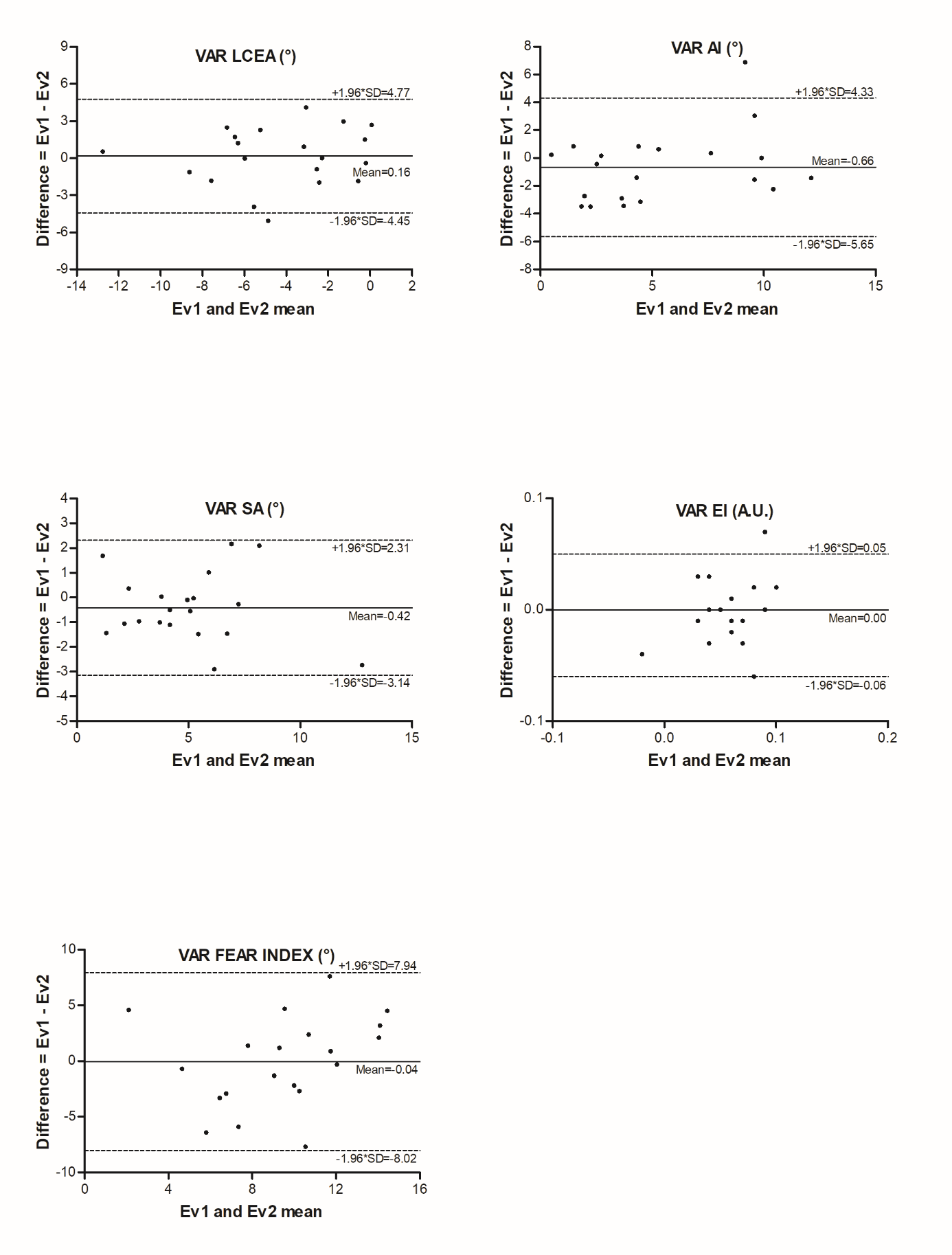


LCEA: lateral center-edge angle; AI: acetabular index; SA: sharp angle; EI: extrusion index; VAR: variation; Ev1: Rater 1; Ev2: Rater 2.

**Figure 4.** Inter-Rater Analyses (Second Assessment) for the Non-Manipulated Parameters


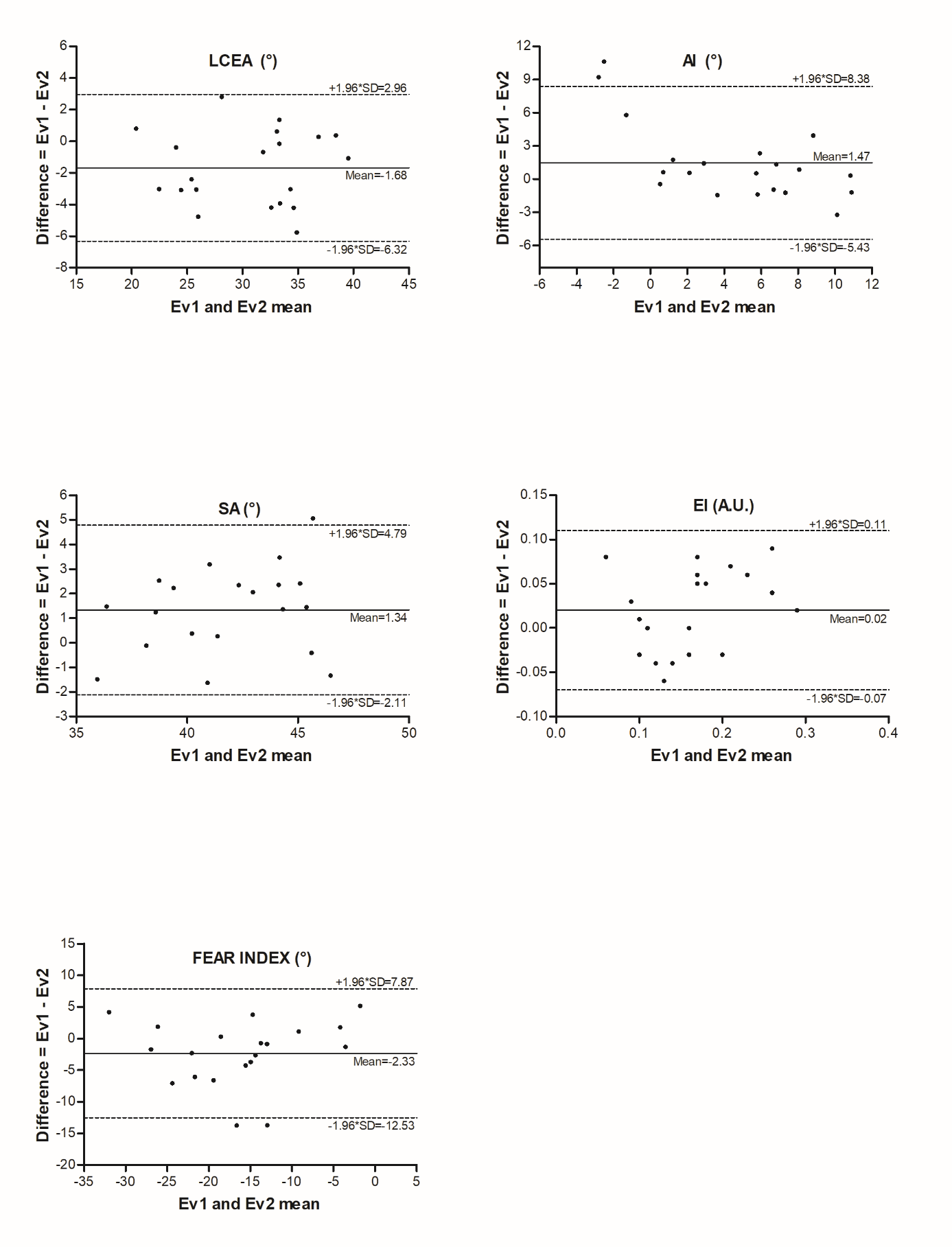


LCEA: lateral center-edge angle; AI: acetabular index; SA: sharp angle; EI: extrusion index; Ev1: Rater 1; Ev2: Rater 2.

**Figure 5.** Inter-Rater Analyses (Second Assessment) for the Manipulated Parameters


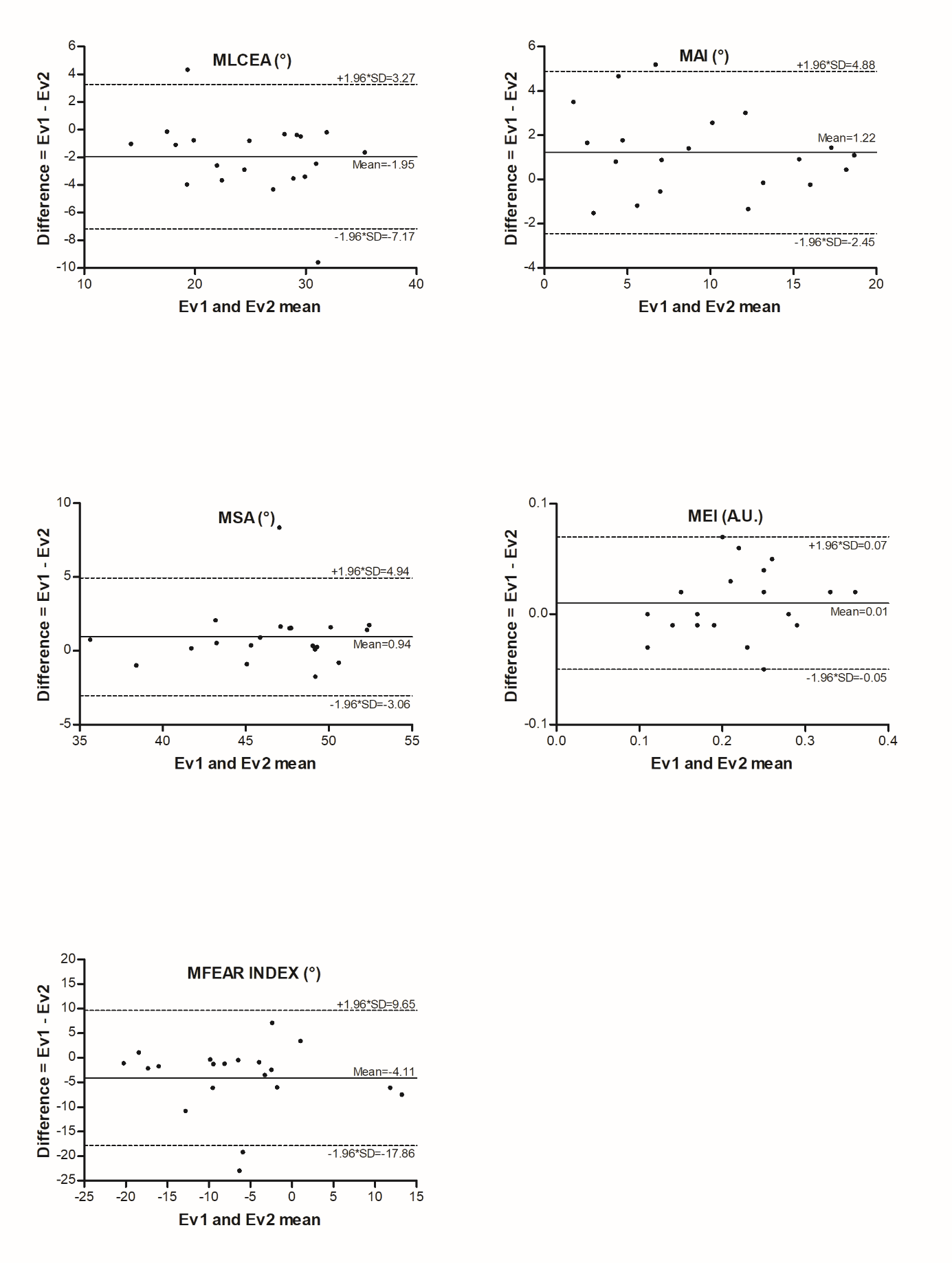


LCEA: lateral center-edge angle; AI: acetabular index; SA: sharp angle; EI: extrusion index; M: manipulated; Ev1: Rater 1; Ev2: Rater 2.

**Figure 6.** Inter-Rater Analyses (Second Assessment) for the Variation


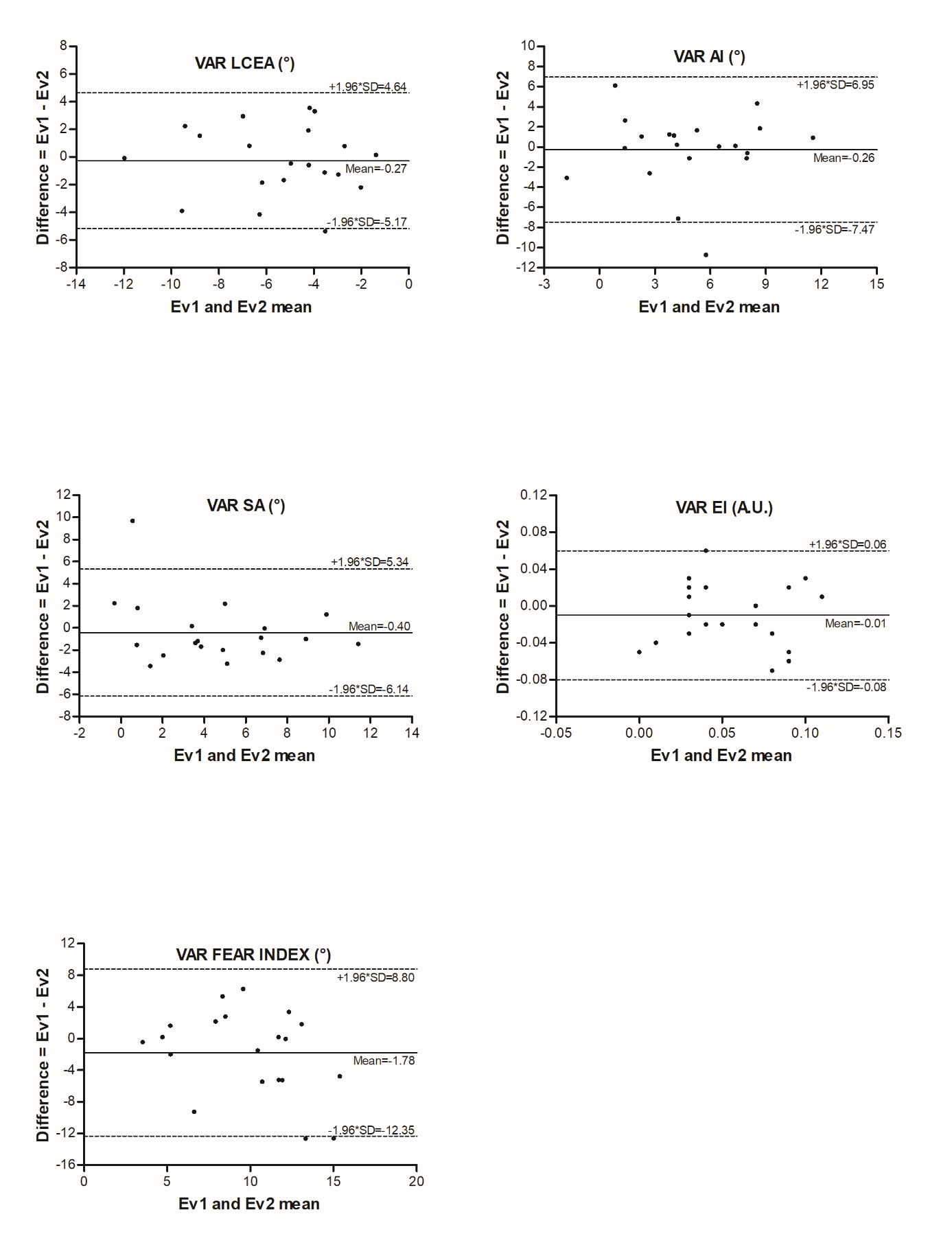


LCEA: lateral center-edge angle; AI: acetabular index; SA: sharp angle; EI: extrusion index; VAR: variation; Ev1: Rater 1; Ev2: Rater 2.

**Figure 7.** Intra-Rater Analyses (Rater 1) for the Non-Manipulated Parameters


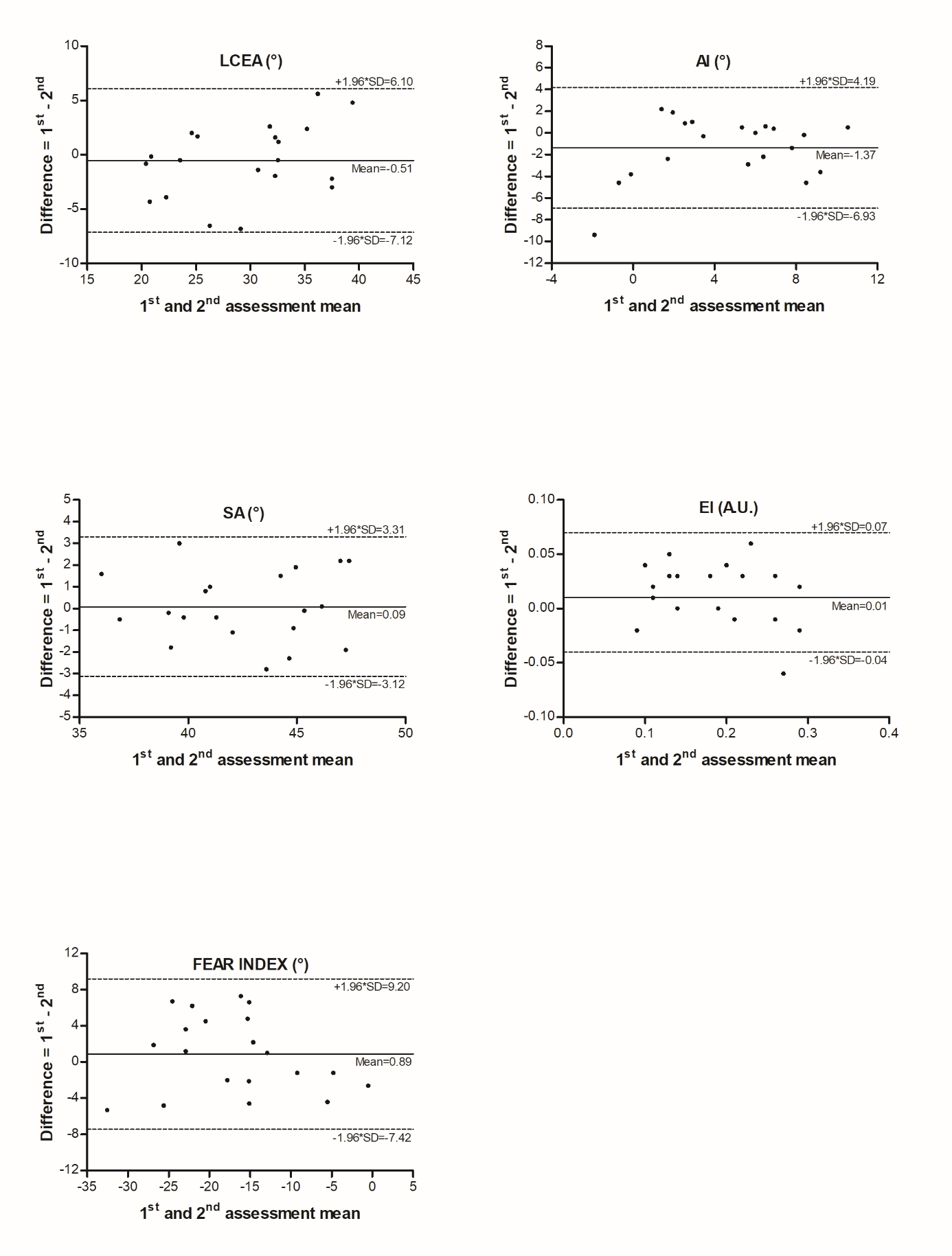


LCEA: lateral center-edge angle; AI: acetabular index; SA: sharp angle; EI: extrusion index.

**Figure 8.** Intra-Rater Analyses (Rater 1) for the Manipulated Parameters


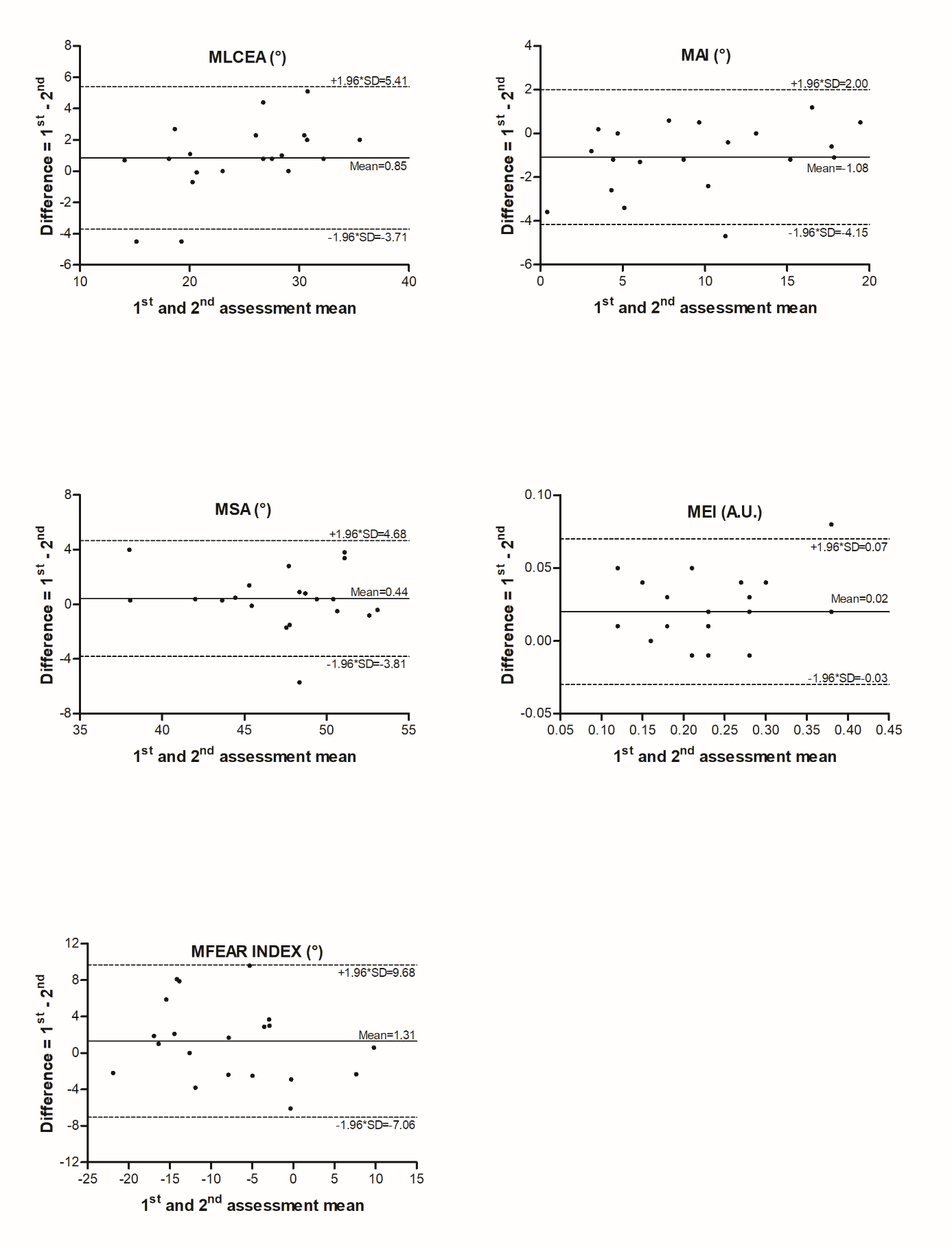


LCEA: lateral center-edge angle; AI: acetabular index; SA: sharp angle; EI: extrusion index; M: manipulated.

**Figure 9.** Intra-Rater Analyses (Rater 1) for the Variation


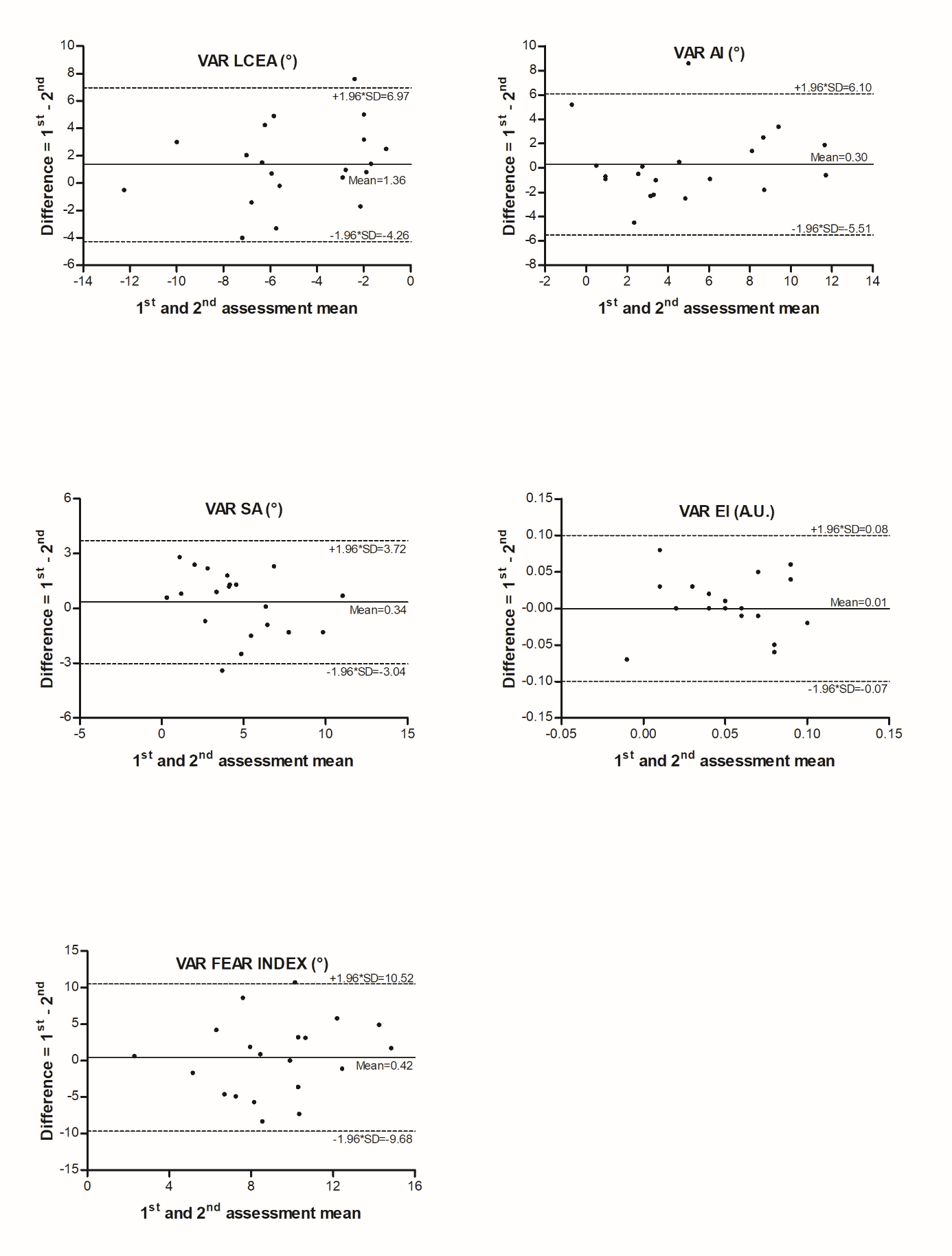


LCEA: lateral center-edge angle; AI: acetabular index; SA: sharp angle; EI: extrusion index; VAR: variation.

**Figure 10.** Intra-Rater Analyses (Rater 2) for the Non-Manipulated Parameters


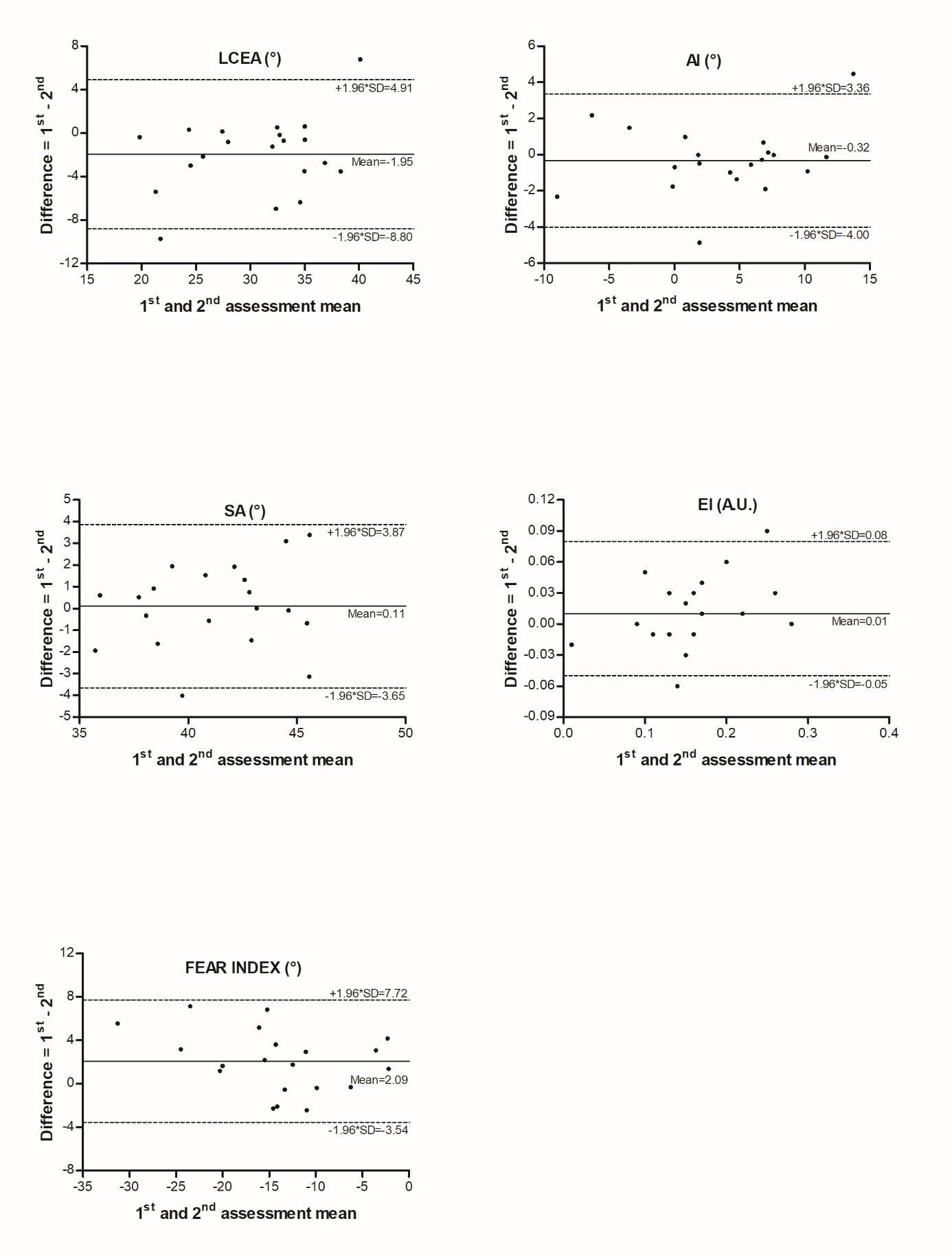


LCEA: lateral center-edge angle; AI: acetabular index; SA: sharp angle; EI: extrusion index.

**Figure 11.** Intra-Rater Analyses (Rater 2) for the Manipulated Parameters


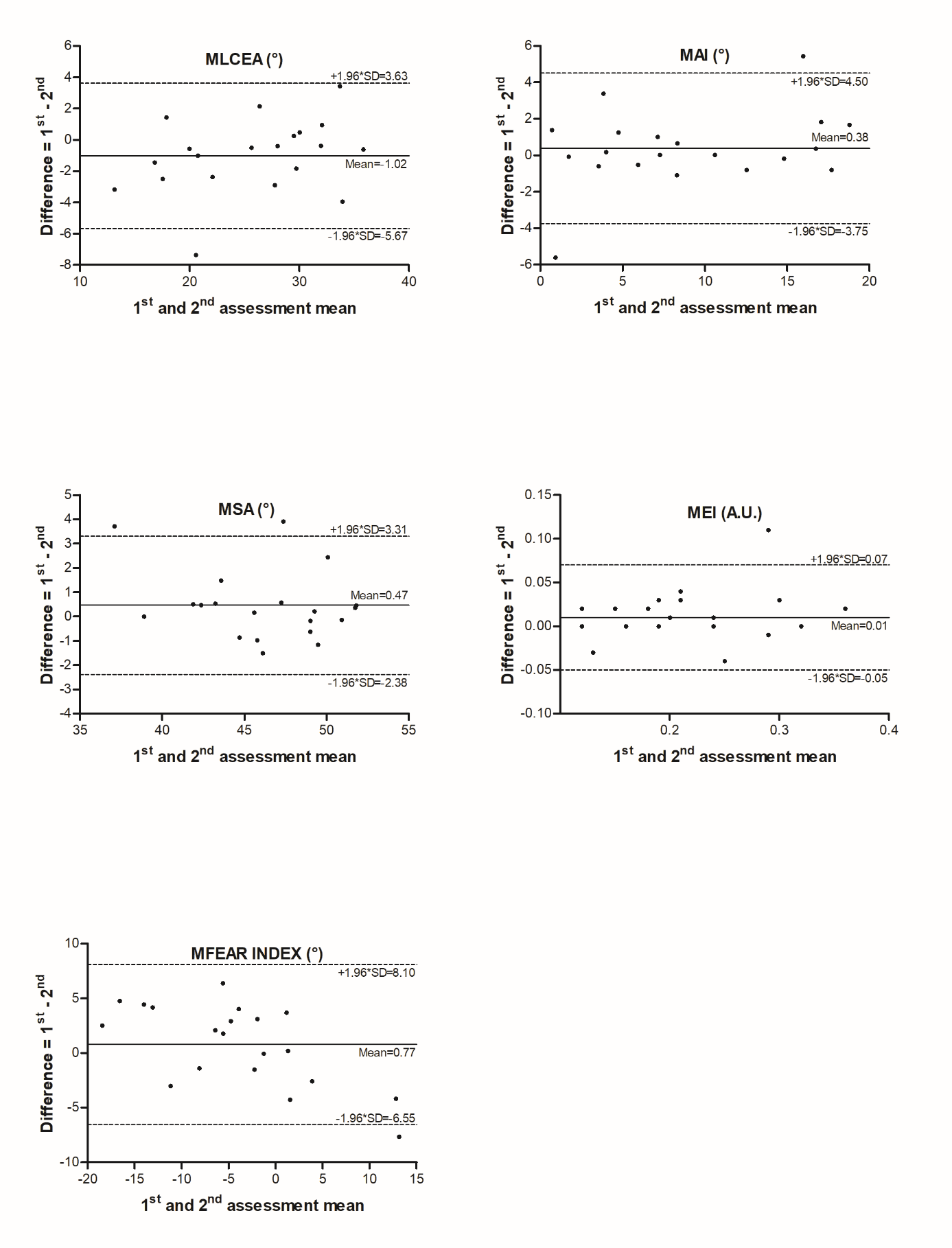


LCEA: lateral center-edge angle; AI: acetabular index; SA: sharp angle; EI: extrusion index; M: manipulated.

**Figure 12.** Intra-Rater Analyses (Rater 2) for the Variation


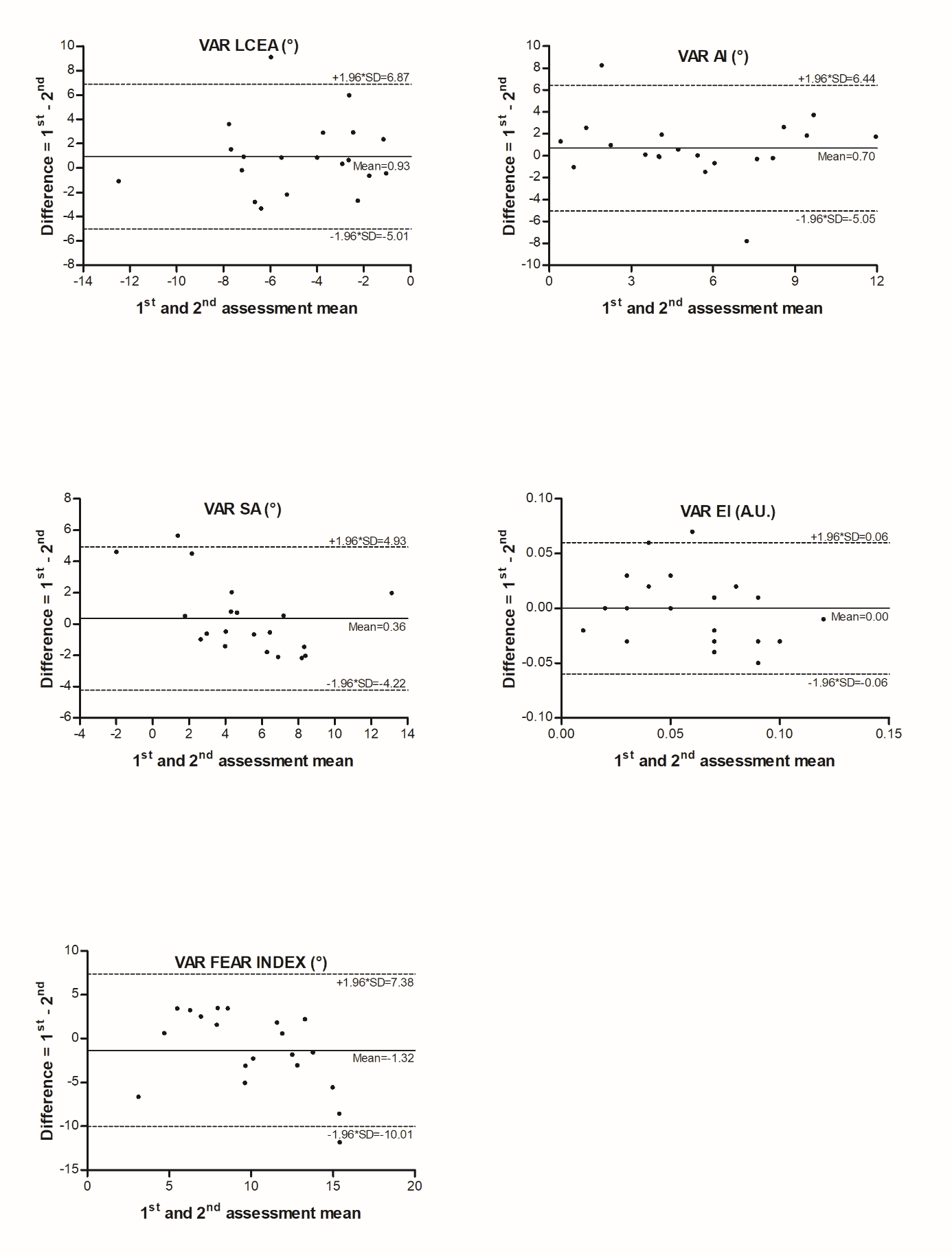


LCEA: lateral center-edge angle; AI: acetabular index; SA: sharp angle; EI: extrusion index; VAR: variation.
